# Can guided self-help for perfectionism improve disordered eating?

**DOI:** 10.1186/2050-2974-1-S1-O21

**Published:** 2013-11-14

**Authors:** Anne O'Shea, Tracey Wade

**Affiliations:** 1 Flinders Univeristy, Australia

## 

Targeting perfectionism in psychological treatment has shown to be effective in improving negative affect, one of the two most potent risk factors for eating disorders (Fittig & Jacobi, 2010), and decreasing symptomology of disordered eating.

The study aimed to evaluate the efficacy of a guided self-help intervention focussed on perfectionism to improve negative affect and the symptoms of disordered eating, anxiety and depression. Participants were assessed at five time points, creating a four-week control and a four-week information-only condition prior to the commencement of eight treatment sessions, and three-month follow-up. All were above the community mean for perfectionism. Of the 33 participants that completed the first three waves (baseline period), 21 participants (64%) reported symptoms of disordered eating on at least one assessment. The presentation will focus on this clinical subsample. Nineteen participants reported disordered eating symptomology over the baseline period. Of the 14 that completed treatment, eight no longer met criteria for an eating disorder at post-treatment, however two failed to maintain this improvement to follow-up. Changes in severity and comorbidities will be discussed along with investigations of the outcome variables, negative affect and weight and shape concern. The presentation will provide results comparing pre-and post-intervention and follow-up assessments.

This abstract was presented in the **Disordered Eating – Characteristics & Treatment** stream of the 2013 ANZAED Conference.

